# Immune modulation mimics damage control orthopaedics’ upregulation of anti-inflammatory miRNA-21/23a/27a and miRNA-30b in the lung after polytrauma in pigs

**DOI:** 10.1007/s00068-025-03005-3

**Published:** 2025-11-27

**Authors:** Belinda Freiin von Münchhausen, Rald V. M. Groven, Carlos J. Peniche Silva, Johannes Greven, Ümit Mert, Tom Eirik Mollnes, Markus Huber-Lang, Klemens Horst, Frank Hildebrand, Martijn van Griensven, Elizabeth R. Balmayor

**Affiliations:** 1 Department of Cell Biology-Inspired Tissue Engineering, MERLN Institute for Technology-Inspired Regenerative Medicine, Universiteitssingel 40, 6229 ER Maastricht, The Netherlands; 2 Experimental Orthopaedics and Trauma Surgery, RWTH Aachen University Hospital, Pauwelsstraße 30, 52074 Aachen, Germany; 3 Department of Orthopedic, Trauma- and Reconstructive Surgery, RWTH Aachen University Hospital, Pauwelsstraße 30, 52074 Aachen, Germany; 4 Research Laboratory, Nordland Hospital Bodø, Parkveien 95, 8005 Bodø, Norway; 5 Department of Immunology, Oslo University Hospital and University of Oslo, Sognsvannsveien 20, 0372 Oslo, Norway; 6 Institute of Clinical and Experimental Trauma Immunology, University Hospital Ulm, Helmholzstraße 8/1, 89081 Ulm, Germany

**Keywords:** Multiple trauma, MicroRNAs, ARDS, Complement inhibition, Early total care, Damage control orthopaedics

## Abstract

**Purpose:**

Blunt chest trauma is common in polytraumatised patients and often leads to respiratory distress. Moreover, the systemic inflammation resulting from the trauma itself, along with subsequent surgical interventions, further contributes to pulmonary dysfunction. Therefore, modulating post-traumatic immune responses may offer potential benefits. MicroRNAs may influence the activation and progression of regenerative responses following polytrauma and could serve as potential modulators. This study investigates the expression of a selection of miRNAs with known involvement in pulmonary pathologies relevant for the post-trauma setting, in a porcine polytrauma model comparing two surgical treatment groups and one treatment group that additionally received a drug-based treatment based on combined inhibition of complement component C5 and the Toll-like co-receptor CD14.

**Methods:**

The porcine polytrauma model consisted of blunt chest trauma, bilateral femur fractures, liver laceration, and haemorrhagic shock. Four groups were defined: sham, early total care (ETC: *n* = 8), damage control orthopaedics (DCO: *n* = 8), ETC with C5/CD14 inhibition (*n* = 4). Animals were monitored and guideline-treated in an ICU setting for 72 h. After sacrifice, lung samples were taken from the left lobe. MiRNAs were analysed by qPCR. Furthermore, Periodic Acid Schiff staining and *in situ* hybridisation were performed.

**Results:**

MiRNAs associated with lung function, inflammation, and fibrosis were analysed. Compared to ETC, DCO resulted in less inflammatory and fibrotic miRNA expression, consistent with histological findings showing more preserved alveoli, less septal thickening, and fewer inflammatory cell infiltrations. The addition of C5/CD14 inhibitors to ETC further reduced the expression of inflammatory and fibrotic microRNAs compared to both DCO and ETC and revealed a significant reduction in histopathological changes in the lung tissue.

**Conclusion:**

This study indicates that combined inhibition of C5 and CD14 effectively reduces posttraumatic histopathological changes in lung tissue associated with less inflammatory and fibrotic miRNA expression, compared to both the DCO and ETC groups.

## Background

Blunt chest trauma is among the most prevalent injuries in polytraumatised patients, and accounts for 20 to 25% of all trauma related deaths [1, 2]. The focused assessment of chest trauma has therefore been a cornerstone of the Advanced Trauma Life Support guidelines since their introduction, dating back to 1978 [3]. In the polytraumatised patient, chest trauma is a frequent severe injury contributing to a certain degree of local but also systemic inflammation. On top of that, patients often require surgery after polytrauma, which further exacerbates and/or reactivates inflammatory responses, a phenomenon commonly referred to as ‘’the second hit’’ [4]. Potential subsequent post-traumatic (immune) complications, such as acute respiratory distress syndrome (ARDS) and the systemic inflammatory response syndrome can be detrimental to patient recovery [5]. Regarding the lungs, it is known that surgical invasiveness, and in particular intramedullary nailing of long bones, can contribute significantly to the occurrence of pulmonary complications in the early post-traumatic phase, like ARDS and pulmonary fat embolisms [6]. These complications are partly responsible for the high mortality rates associated with blunt chest trauma and form the basis of various trauma management protocols, such as Early Total Care (ETC) and Damage Control Orthopaedics (DCO). The patient's immune status, as represented by various standard physiological and biochemical parameters, guides the timing and type of surgery after trauma [7]. Although not implemented in the clinical routine so far, therapeutic immune modulation after trauma has therefore gained interest over the years to reduce or prevent these complications. It may also lessen the effects of early definitive fracture fixation, e.g. by intramedullary nailing, and thereby facilitate its administration even to a more critically ill patient population [8]. Of particular importance is the regulation of the innate part of the immune system, which is the main player in the early post-traumatic phase [4].

Previous studies have already shown that blocking the fluid phase part of the innate immune response, the complement system, can help to improve both immune and organ function after trauma and/or haemorrhagic shock as shown when blocking the central complement component C3 [8, 9]. Further downstream blockade, on the C5 level, also revealed some organ-protective effects during systemic inflammatory conditions [10–14]. Moreover, complement inhibition combined with a toll-like receptor blocker (e.g., by the C5 inhibitor and anti-CD14) has proven effective in *in vivo* sepsis models, but research on its potential role in trauma treatment is lacking [15, 16]. Adding such a drug combination may prevent the onset of (immune) pulmonary complications after trauma and could allow for earlier definitive fracture fixation (ETC).

At a biomolecular level, these (patho-)physiological responses to trauma, surgery, and the C5/CD14 inhibition are regulated by cellular communication and signalling, which in great part depend on the expression of microRNAs (miRNAs). These small non-coding RNA molecules regulate protein expression by interfering with messenger RNA (mRNA) translation or enhancing promotor activity of a specific gene [17]. Research has already shown that miRNAs are important in several pulmonary pathologies, such as lung cancer, fibrosis, and chronic respiratory diseases [18]. Furthermore, several studies have shown that specific post-traumatic miRNA expression patterns in both injured tissues and in the systemic circulation correlate with patient specific parameters, trauma severity, and (organ specific) complications [17].

Based on previous findings, we hypothesize that miRNAs play an important regulatory role in the occurrence of pulmonary complications in the acute post-traumatic phase. Unfortunately, research on the exact role of miRNAs in relation to pulmonary complications after polytrauma is scarce. Furthermore, possible correlations of miRNA expression with treatments, being those of drug or surgical nature, have not been investigated.

In this study we aimed to investigate the expression of a selection of miRNAs with known involvement in pulmonary pathologies in the trauma setting, in a porcine polytrauma model comparing two surgical treatment methods (i.e., ETC and DCO), as well as one separate ETC treatment group receiving C5/CD14 inhibition (drug-based treatment). In addition, we performed bioinformatic analyses to predict mRNA targets of the deregulated miRNAs and gained insights into the implicated biological processes and pathways. Lasty, we investigated the tissue/cell distribution and abundance of specific miRNAs in the pulmonary parenchyma by means of *in situ* hybridisation.

## Methods

### Animal ethics, care, and group allocation

The present data were gathered as part of a larger study according to the principles of replacement, refinement, and reduction of animal research [19]. The present study was designed *a priori*. The German government’s Office of Animal Care and Use (Landesamt für Natur, Umwelt und Verbraucherschutz; LANUV, Nordrhein-Westfalen, Recklinghausen, Germany) approved the study under permit number 81–02.04.2020.A215. All sections of this manuscript have been written based on the ARRIVE guidelines for reporting animal research [20].

In total, 26 German Landrace pigs (male, *Sus scrofa*), aged three months and with 35 ± 5 kg of bodyweight, were used for this study. A veterinarian clinically examined all animals upon arrival at the animal facility. Animals were housed in ventilated rooms for seven days before the start of the study for acclimatization purposes. Animals were randomly divided into either the control group or one of the three treatment groups investigated in the study. A sham group (*n* = 6) served as control, and received identical instrumentation, anaesthesia, mechanical ventilation, fluid administration, and nutrition throughout the study. Out of the three study groups, two included surgical treatments. A total of 16 animals were equally divided into two surgical treatment groups of either ETC (*n* = 8) or DCO (*n* = 8). The drug treated group received a combination of a C5 and a CD14 inhibitor at several timepoints after trauma, as described in detail below, combined with the regular ETC treatment (*n* = 4).

### Polytrauma induction and resuscitation

Animals were fasted for 12 hours (h) with *ad libitum* water before instrumentation, drug administration, and trauma application. Premedication, anaesthesia, fluid administration and nutrition, as well as instrumentation are described in detail in Table 1 and Table 2, and it has been extensively described elsewhere [19, 21].

**Table 1:**
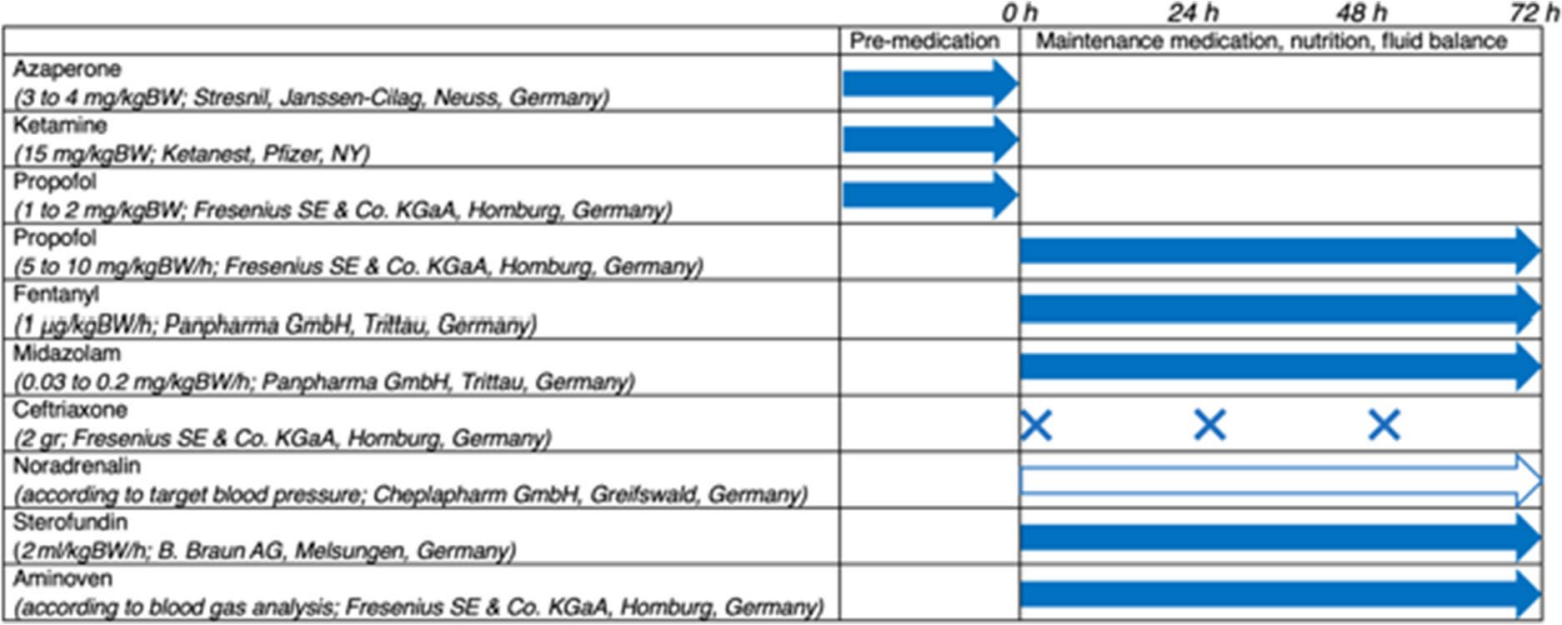
Overview of premedication, anaesthesia, vasopressor therapy, and fluid and nutrition administration

Blue arrows indicate a single dose for premedication, and a continuous infusion or perfusion for as long as the study duration, 72 h. Blue crosses represent the single administration of an antibiotic. White arrow indicates vasopressor, which was given on demand based on the mean arterial blood pressure

**Table 2:** Overview of the instrumentation prior to trauma induction

Catheter	Manufacturer
Central venous catheter	Teleflex Medical GmbH, Fellbach, Germany
Vena Jugularis externa, 4-lumen, 8.5 Fr	
Hemodialysis catheter	Teleflex Medical GmbH, Fellbach, Germany
Vena Femoralis, 3-lumen, 12.0 Fr	
Arterial catheter	Vygon GmbH & Co. KG, Aachen, Germany
Arteria Femoralis	
Suprapubic urinary catheter	B. Braun AG, Melsungen, Germany
Mini-laparotomy, 12.0 Fr	

After orotracheal intubation, mechanical, volume-controlled ventilation was performed (8–12 ml/kgBW); Positive End-Expiratory Pressure 8 mmHg (plateau pressure < 28 mmHg) adjusted by capnometry (pCO_2_ of 35–45 mmHg) (Draeger Evita 4, Draeger Safety AG & Co. KGaA, Luebeck, Germany). The fraction of inspired oxygen (FiO_2_) was kept at 0.21 throughout instrumentation, trauma induction, and the subsequent shock phase to mimic ambient air.

For all animals, body temperature, blood pressure (including mean arterial pressure, MAP), heart and respiratory rates, electrocardiogram recordings, and electrocardiogram-synchronized pulse oximetry were continuously monitored. Throughout the study, the sham group received identical instrumentation, anaesthesia, mechanical ventilation, fluid administration, nutrition, and vasopressor therapy, if required (Tables 1 and 2).

Trauma was induced after maintenance of stable baseline parameters for at least 120 min. The animals were then subjected to the standardized polytrauma (injury severity score = 27), consisting of blunt chest trauma, bilateral femur fractures, sharp abdominal trauma, and haemorrhagic shock, as follows. The blunt chest trauma was induced by firing a Blitz-Kerner bolt gun machine (Turbocut JOPP GmbH, Bad Neustadt an der Saale, Germany) with cattle killing cartridges (9 × 17 mm; DynamitNobel AG, Troisdorf, Germany) on a pair of steel and lead plates (8 and 10 mm, respectively). The pair of steel and lead plates was placed on the right dorsal, lower hemithorax, after which an inspiratory hold upon full inspiration was performed. Immediately after establishment of the inspiratory hold, the bolt gun machine was fired onto the plates. To induce the bilateral femur fractures, the bolt gun was fired on a custom-made punch located on the middle third of the femora. The liver laceration was simulated by a midline laparotomy and transverse incisions (4.5 × 4.5 cm) halfway through the liver tissue in the left lobe. After 30 s of uncontrolled bleeding, five sterile 10 × 10 cm gauzes were used for hepatic packing. In addition, blood was withdrawn in citrate phosphate dextrose adenine blood bags (DONOpacks, Lmb Technologie GmbH, Oberding, Germany) until the MAP reached 40 ± 5 mmHg (i.e., to a maximum of 45% of total blood volume) and fluid administration was reduced to 0.1 ml/kgBW/h to keep the infusion lines open for later use. No measures were taken to prevent hypothermia. Similarly, no vasopressor therapy was provided throughout the following 90 min shock phase to mimic a real-life scenario.

Resuscitation was performed after 90 min and according to the guideline on Treatment of Patients with Severe and Multiple Injuries®, ATLS® and AWMF-S3 [22]. The FiO_2_ was adjusted to base values, the withdrawn blood was re-infused (DONOpacks), and fluid administration was resumed (Table 1). Avoidance of hypothermia was achieved (porcine normothermia: 38.7–39.8°C) with a forced-air warming system and blankets.

The animals were monitored and treated in a veterinary intensive care unit for 72 h after trauma induction and received antibiotic infusions (ceftriaxone) before surgery and every subsequent 24 h for the duration of the study. Furthermore, animals were turned every four to six hours to prevent thrombosis and support the breathing mechanics of the animals. Animals were sacrificed at 72 h.

### Experimental treatments

After the shock phase (90 min), the animals were operatively and medically stabilized. The eight pigs assigned to the ETC group received an intramedullary nail (T2 System, Stryker GmbH & Co. KG, Duisburg, Germany) for both femoral fractures. The additional set of eight pigs allocated in the DCO group received external fixators (Radiolucent Fixator, Orthofix, Texas, USA) for both femur fractures. The third experimental group consisting of four pigs received ETC combined with the C5/CD14 inhibition therapy. The drugs were administered intravenously to the animals at 30 min after polytrauma induction. The C5 inhibitor, RA101295 (2-kDa peptide; UCB, Brussels, Belgium) and the recombinant anti-porcine CD14 antibody rMil2 (clone MIL2; isotype IgG2a; ExcellGene Sa, Monthey, Switzerland) were used. A pilot animal was used to determine the effective dosage of the C5 and CD14 inhibition drugs in pigs. For this, boluses of the inhibition drugs were given (i.e., 3 mg/kgBW of C5 and 5 mg/kgBW of CD14) followed by a continuous infusion of the C5 inhibitor at 0.55 mg/kgBW/h until 64 h after trauma. The obtained results from the pilot dosage finding experiment, reported in detail by Lupu et al. [19] allowed us to set up the following drug treatment regimen for the following *n* = 3 animals in the ETC + C5/CD14 group, 5 mg C5 inhibitor drug per kgBW was administered at 30 min after trauma, followed by a continuous infusion of 1.1 mg/kgBW/h until 72 h after trauma. In addition, the CD14 inhibition drug was administered using 5 mg/kgBW at 30 min, 12 h, and 30 h after trauma. Thereafter, the dosage was reduced to 2.5 mg/kgBW and applied at 60 h after trauma. The four animals were grouped together as there was no variability in treatment effect between the first, pilot evaluation animal and the other three animals.

### Tissue sample harvest, RNA isolation, and cDNA synthesis

After 72 h, the animals were sacrificed and lung samples from all four groups, sham + 3 experimental groups were collected from the right upper lobe. Samples were snap frozen in microcentrifuge tubes (Biotix, San Diego, CA, USA) using liquid nitrogen and transferred to −80°C. Samples were lysed in Trizol reagent (Thermo Fisher Scientific, Waltham, MA, USA) using a Qiagen Tissue Lyser LT (Qiagen, Venlo, The Netherlands) for 5 min at 50 oscillations after which the homogenate was either directly used for RNA isolation, or stored at −80°C for later use. Chlorophorm phenol RNA isolation was performed using GlycoBlue (Thermo Fisher Scientific, Waltham, MA, USA) as co-precipitant according to the manufacturer’s instructions. The RNA isolates were analysed for quantity and purity using spectrophotometry (Biodrop µLite +, Biochrom, Holliston, MA, USA). Thresholds for A260/A230 (≥ 1.7) and A260/A280 (≥ 1.8) ratios were used for RNA sample quality assessment. The Monarch® RNA Cleanup Kit (New England Biolabs, Ipswich, MA, USA) was applied for RNA purification when necessary. The miRCURY LNA RT Kit (Qiagen, Venlo, The Netherlands) was used to transcribe 40 ng of template RNA for each individual sample into cDNA following the manufacturer's instructions.

### miRNA expression

Twelve miRNAs, associated with pulmonary contusion and inflammation, ARDS, and (blunt)chest trauma were selected for study. The selected miRNAs were miR-19a-3p, −21-5p, −23a-3p, −27a-3p, −30b-3p, −34a-5p, −126a-3p, −146a-3p, −150-3p, −155-5p, −181a-5p, and −223-3p. The expression of each of this miRNA was evaluated in lung tissue samples from individual each animal corresponding to all *n* = 4 included groups. The miRNA expression was determined using customized Qiagen (Qiagen, Venlo, The Netherlands) miRCURY qPCR primer assays. A list containing the Gene Globe identification numbers of the respective primers is provided in the Dataverse repository (10.34894/TIWYZE). The miRNA expression levels were assessed by the quantification cycle (C_q_) value using a CFX96 real-time quantitative PCR system (Bio-Rad, Munich, Germany). Two housekeeping genes, namely miR-103a-3p and U6snRNA were tested. U6snRNA was selected based on performance and obtained results. An upregulation was represented by the fold regulation value as calculated by the 2^−ΔΔCt^ method, while a downregulation was represented by the negative inverse of the calculated 2^−ΔΔCt^, fold regulation values of ≥ 1.5 or ≤ −1.5 were considered significant.

### mRNA target prediction

An extensive literature review was conducted to identify mRNAs of interest related to (pulmonary) inflammation, pulmonary contusion, endothelial dysfunction, and leukocyte infiltration. Cellular signalling pathways involved in survival, proliferation, and cellular communication, as well as tissue remodelling were considered. The miRanda Target Scanner algorithm version 3.3a (http://www.microrna.org) was used to predict potential targets. This algorithm considers sequence complementarity based on positive-weighted local alignment, which evaluates miRNA-mRNA sequence matching, and free energy of the duplex structure to measure the stability. It also examines interspecies sequence conservation. The predicted miRNA-mRNA interactions were visualized using Cytoscape 3.10.0 (http://cytoscape.org).

### Function of predicted genes

Functional enrichment analyses (Gene Ontology; GO) were performed on the predicted target genes using the ShinyGO bioinformatic platform (ShinyGO 0.80; http://bioinformatics.sdstate.edu/go/). Significantly enriched GO terms signify a biological process, cellular component, or molecular function in which a substantial proportion of the predicted mRNA targets is implicated. The enrichment score, representing the degree of over-representation of a specific GO term in the target gene set compared to the reference set, was typically determined as the negative logarithm of the corresponding false discovery rate (FDR; cut-off 0.05).

### Periodic acid schiff (PAS) staining

Lung sections were stained following the IHC World staining protocol (IHC World LLC., Woodstock, NY, USA). In short, the samples were deparaffinized, oxidized in 0.5% periodic acid solution, and rinsed with distilled water. The slides were placed in Schiff reagent for 15 min after which they were rinsed in lukewarm water for 5 min. A counterstain with Mayer’s haematoxylin for 1 min was used, after which the samples were washed with water for 5 min, dehydrated and mounted with Ultrakitt (J.T. Baker, Phillipsburg, NJ, USA). Images were captured using a Fritz slide scanner (Precipoint GmbH, Garnich, Germany).

### In situ hybridisation of specific miRNAs

Based on the obtained miRNA expression results, *in situ* hybridisation (ISH) was performed for the most up- and downregulated miRNAs, i.e., of miR-30b-3p and −126a-3p, respectively. The ISH experiments were executed in RNAse-free conditions, according to the manufacturer’s protocols. Lung tissue was embedded in paraffin, cooled to −5°C using a EG1130 Cold Plate (Leica Biosystems, Wetzlar, Germany) and sectioned 5–8 µm thick using a microtome (RM2035 BioCut, Leica Biosystems, Wetzlar, Germany). *In situ* hybridisation was performed using Qiagen’s double-DIG miRNA detection probes for miR-30b-3p and −126a-3p following the miRCURY LNA miRNA detection kit protocol (Qiagen, Venlo, The Netherlands). A scramble probe and the U6 snRNA detection probe were used as negative and positive control respectively.

In short, the samples were deparaffinized and rehydrated using NeoClear-xylene substitute, descending serial dilutions of ethanol, and nuclease-free water (Qiagen, Venlo, The Netherlands). Subsequently, samples were incubated for 10 min at 37°C with proteinase K (SigmaAldrich, St. Louis, MO, USA) in a Dako hybridizer (Dako Colorado, Inc., Fort Collins, CO, USA). The slides were then washed twice with sterile PBS (Thermo Fisher Scientific, Waltham, MA, USA) after which they were incubated with the hybridisation mix containing the detection probes or controls for 60 min at 55°C in the Dako hybridizer. The working concentration used was 40 nM for the miRNA detection probes and the scramble probe, and 1 nM for the LNA U6 snRNA detection probe, as recommended by the manufacturer. After hybridisation, the slides were thoroughly washed with 5X, 1X, and 0.2X SSC buffer (Thermo Fisher Scientific, Waltham, MA, USA) at 55°C. Blocking was carried out with 1% BSA (SigmaAldrich, St. Louis, MO, USA) solution for 15 min at room temperature prior to a 1 h incubation with anti-DIG (dilution 1:800). The samples were washed with PBS and incubated for 2 h at 30°C in a humidity chamber with freshly prepared AP substrate. Afterwards, AP-stop solution was applied to the samples followed by a counterstaining with fast red (Merck, Darmstadt, Germany) for 4 min and then washed for 10 s with a 1% acetic acid solution. Lastly, samples were dehydrated in an increasingly concentrated ethanol series before being mounted with UltraKit mounting media (J.T. Baker, Leicestershire, UK). Images were captured using a Fritz slide scanner (Precipoint GmbH, Garnich, Germany).

### Statistical analysis

Statistical analyses were performed using GraphPad Prism version 9.1.1 (GraphPad Software, San Diego, CA, USA). Data are reported as mean with standard deviation or standard error of the mean, as appropriate. Normality of the data was assessed using the Shapiro–Wilk test, after which either a one-way ANOVA or Kruskal–Wallis test was applied, followed by Holm-Šídák's or Dunn’s post-hoc test for multiple comparisons. In this study, a significance level of *p* ≤ 0.05 was considered statistically significant. Significant differences were displayed as * *p* < 0.05, ** *p* < 0.01, and *** *p* < 0.001.

## Results

One animal in the ETC group did not survive the full 72-h observation period of the experiment. It experienced cardiac arrest 60 h after trauma induction and did not respond to immediate resuscitation. Consequently, the collected data from this animal was excluded from the analysis, resulting in *n* = 7 animals included in the ETC group instead of the planned *n* = 8.

### Establishing a miRNA signature of blunt chest trauma

**Fig. 1:**
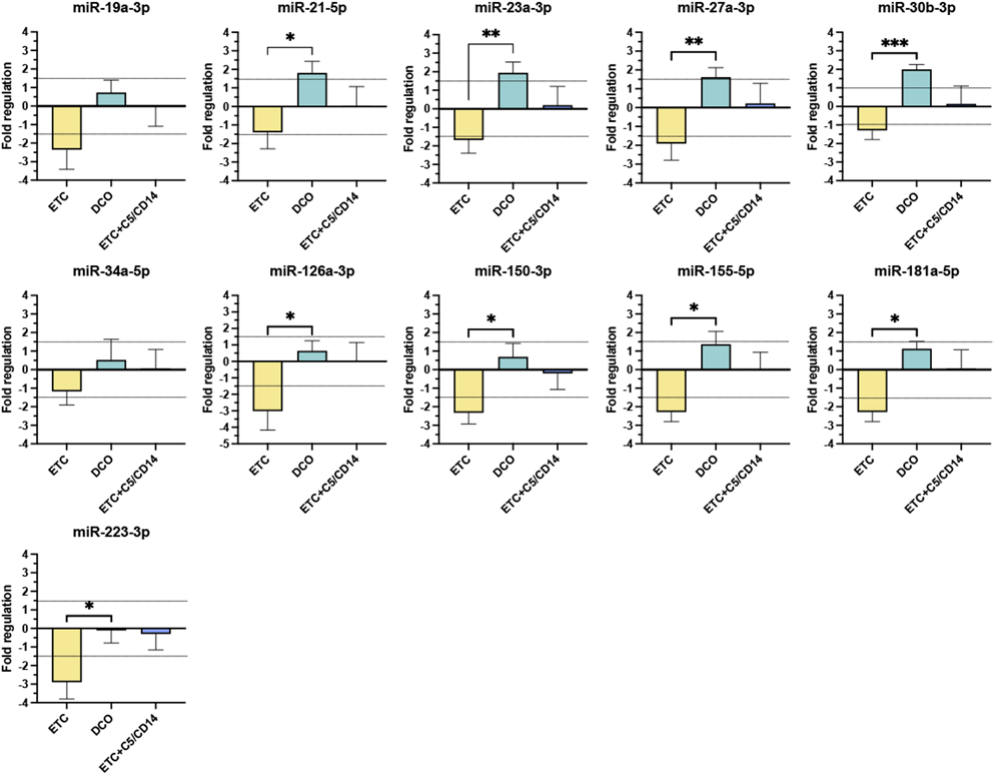
MicroRNAs are differentially expressed among the three treatment groups; Early Total Care (ETC), Damage Control Orthopaedics (DCO), and ETC + C5/CD14 inhibition. Bar charts represent the mean expression level from the specific miRNA, accompanied by the standard error of the mean. Gray dotted lines represent the fold regulation threshold of < −1.5 or > 1.5, below or above which a miRNA was considered significantly deregulated. Significant differences in expression levels between groups are indicated as follows * *p* < 0.05, ** *p* < 0.01, ***p < 0.001

From the 12 evaluated miRNAs, miR-146a-3p, did not show expression in any of the investigated groups. Among the remaining 11 miRNAs, a common trend was observed in which the fold regulation of the ETC group was consistently the lowest, followed by that of the ETC + C5/CD14 inhibition group, and superseded by that of the DCO group (Fig. 1).

Eight out of the 11 detected miRNAs were significantly downregulated in the ETC group, while in the DCO group four of 11 resulted significantly upregulated. The most evident downregulations in the ETC group were miR-126a-3p and miR-223-3p with fold regulation values of −3 and −2.9, respectively. In the DCO group, miR-23a-3p and miR-30b-3p were most upregulated with fold regulation values of 1.9 and 2.0, respectively. Additional treatment with C5/CD14 inhibition drug to the animals that received ETC did not result in significant miRNA deregulation. However, the C5/CD14 inhibition drugs normalized miRNA expression to that of the sham group, as shown by the fold regulation values ranging between −0.3 to 0.2.

### mRNA target predictions

**Fig. 2:**
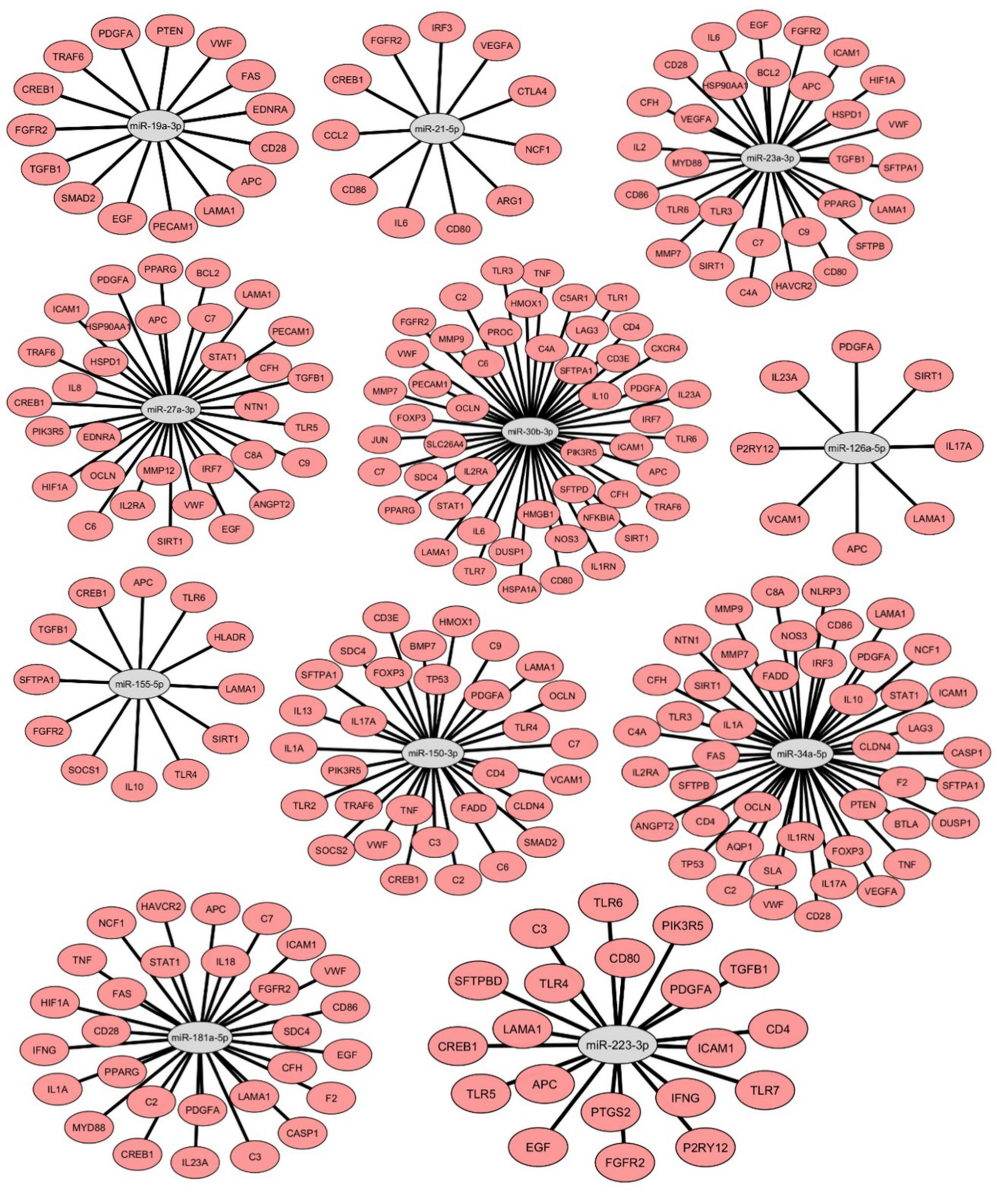
mRNA target prediction results for each miRNA detected in the lung tissue of polytraumatised pigs. Schematic representation generated with Cytoscape. MiRNAs are depicted in grey ovals, surrounded by their predicted mRNA targets

In total, 105 targets were identified for the 11 miRNAs that were confirmed as aberrantly regulated (Fig. 2). These targets were mainly involved in (post-traumatic) inflammation, such as a variety of toll-like receptors and pro- and anti-inflammatory cytokines; the recruitment and activation of immune cells, such as CD-3, −4, and −86; the coagulation and complement cascades, such as von Willebrand factor, protein C and various complement factors; and finally, tissue regeneration, such as fibroblast growth factor receptor 2 and platelet-derived growth factor. Multiple targets related to different aspects of blunt chest trauma overlapped among the different miRNAs. For example, miR-30b-3p, −34a-5p, −150-3p, and −181a-5p showed most miRNA-mRNA interaction matches in terms of inflammation. Furthermore, miR-181a-5p, −30b-3p, and −34a-5p showed the most mRNA interactions concerning the coagulation cascade, and miR-150-3p, −27a-3p, and −30b-3p were most involved in the complement system.

### Bioinformatic analyses

**Fig. 3:**
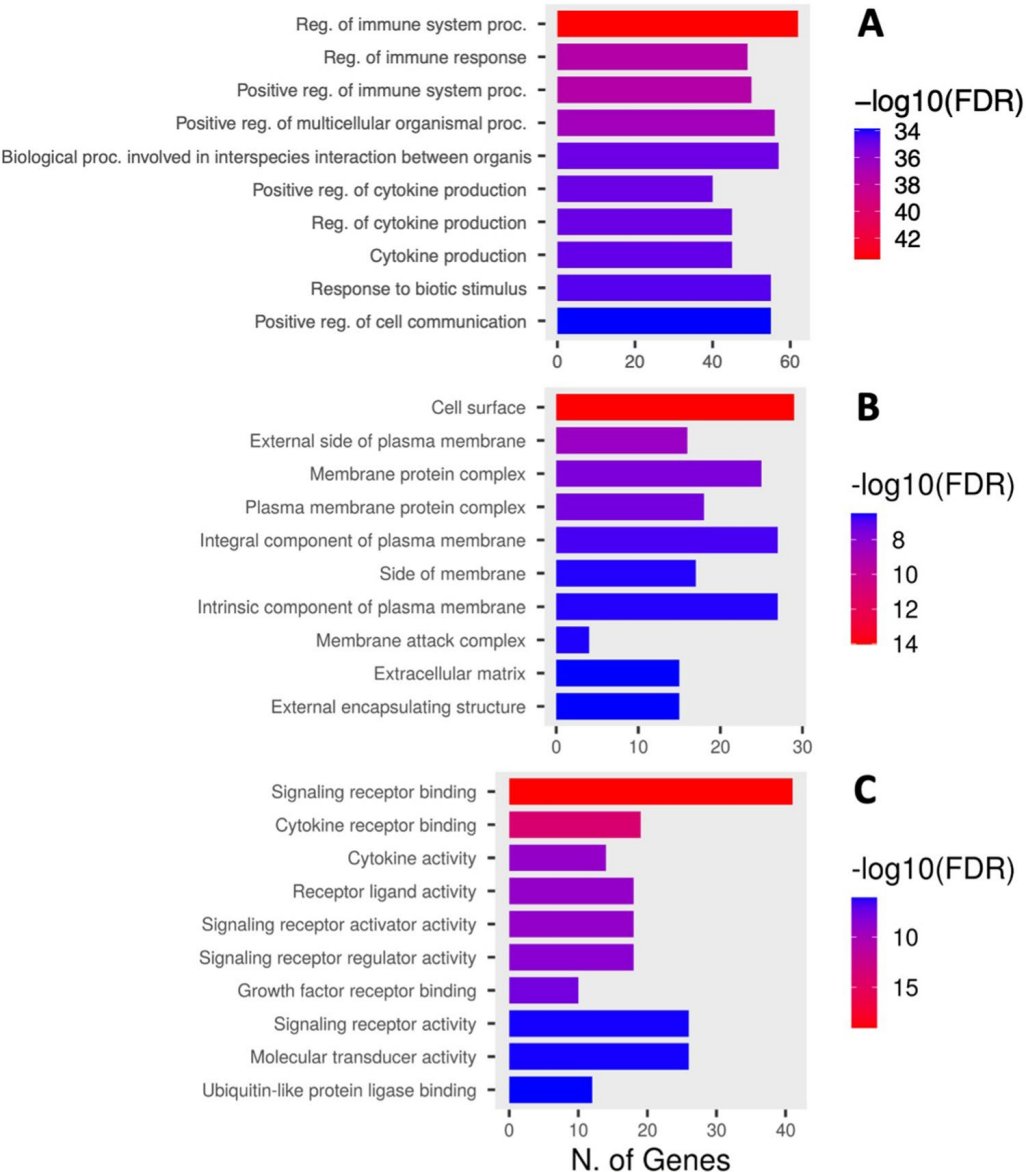
Functional enrichment analyses of the predicted mRNA targets. Only miRNA with significantly deregulation in lung tissue where considered. Significantly enriched gene ontology terms are listed in three categories: (**A**) biological process, (**B**) cellular component, and (**C**) molecular function (ranked by -log_10_(FDR))

Functional enrichment analyses (GO) were conducted to examine the biological significance of the predicted mRNA targets regarding the polytrauma model and the treatment groups. Most significantly enriched terms for each of the three GO categories (i.e., biological process, cellular component, and molecular function) are depicted in Fig. 3. The immune response after trauma was highly represented among the enriched biological process terms (Fig. 3A). Also, the cellular component terms showed several links to the trauma and treatment, such as the membrane attack complex, extracellular matrix, and several plasma membrane components (Fig. 3B). Lastly, the enriched molecular function terms mainly focused on cytokine activity and binding, and ubiquitin-like protein ligase binding (Fig. 3C).

### Lung tissue structure and components as determined by PAS staining

**Fig. 4:**
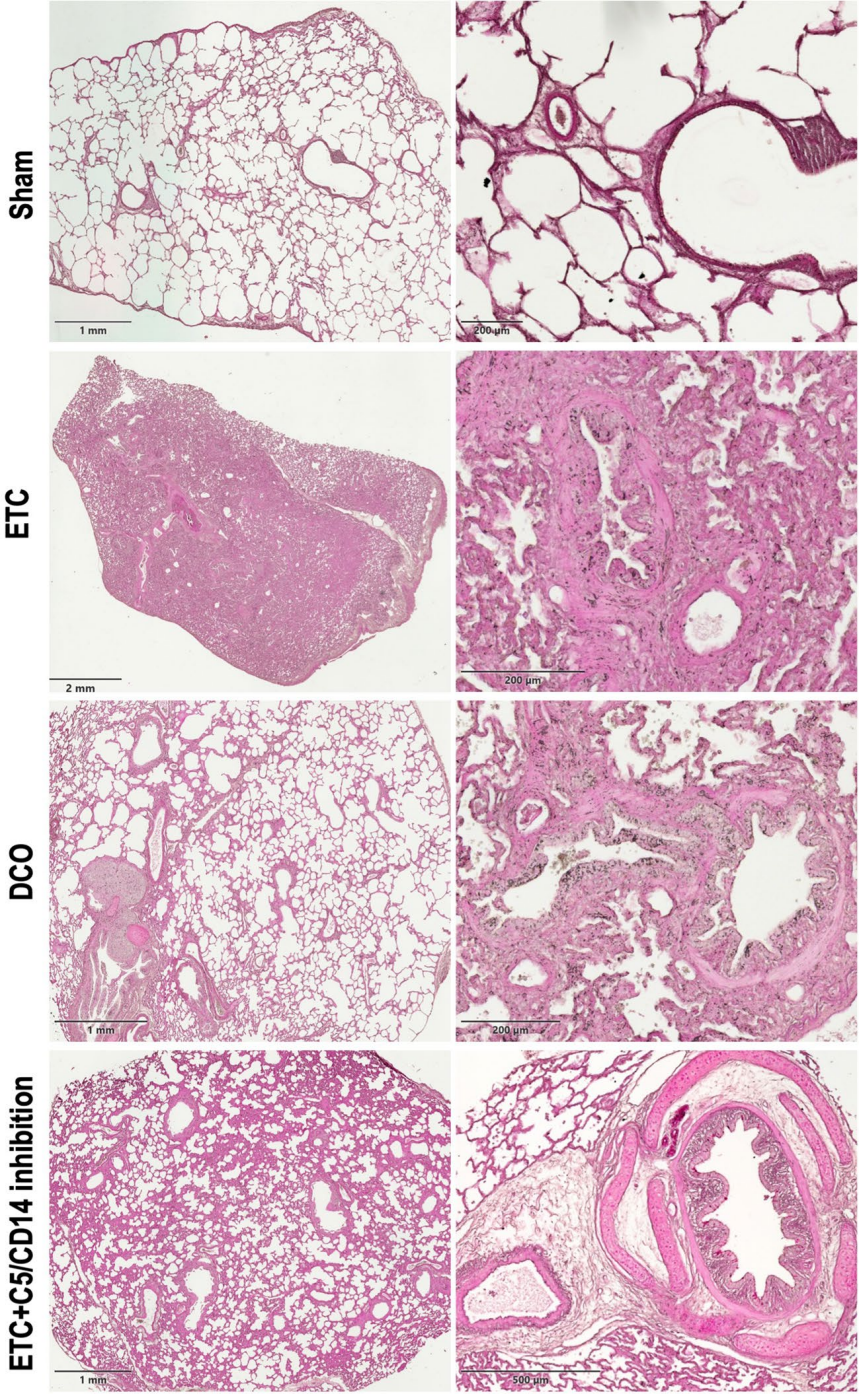
Periodic Acid Schiff (PAS) staining of the investigated groups, i.e., sham, Early Total Care (ETC), Damage Control Orthopaedics (DCO), and combined ETC + C5/CD14 inhibition drugs. Each row depicts two different magnifications per respective treatment group

Tissue sections from the sham group consisted of well-defined, evenly distributed, and unobstructed alveoli with thin and delicate septa. Bronchiole branches were visible, accompanied by vasculature, and peribronchial connective tissue was homogeneously distributed with little to no leukocyte infiltrations. Physiological PAS-positive layers could be identified on the insides of alveoli and beneath epithelial and endothelial bronchiole layers, indicative of surfactant and basement membrane components (Fig. 4).

In the ETC group, the parenchyma showed marked histological changes compared to the sham group. In fact, in the ETC tissue sections, only a few alveoli, bronchioles, and vessels were visible due to excessive inflammation, as seen by the large congestions and infiltrations with inflammatory cells, as well as the formation of hyaline membranes. Diffuse PAS-positive areas were identified in ETC samples, reflecting great quantities of mucin and inflammatory cell secretions, such as glycoproteins, which are critical for tissue repair.

In samples from the DCO group, alveolar, bronchial, and vascular structures delineated in a sharper manner as compared to the ETC group. Also, inflammatory cell infiltrations and interstitial edema were observed, albeit to a lesser degree and more focal in nature as compared to the ETC group. PAS-positive structures were limited to thickened linings in the alveoli and bronchioles, representing increased mucus production. Tissue sections corresponding to the combined therapy group (i.e., ETC + C5/CD14 inhibition drug) showed more diffuse infiltrations of granulocytes and macrophages than those of the DCO group, but less than the ETC group, consistent with the qPCR results. The same applied to the alveoli, bronchioles, and vasculature, which were still largely visible, although the alveolar septa were thickened by interstitial edema and the formation of hyaline membranes. Although to a lesser extent than in the ETC group, the perivascular space and bronchioles stained positive for PAS, indicating increased vascular permeability and excess mucus production, respectively.

### miRNA tissue localisation by ISH

**Fig. 5:**
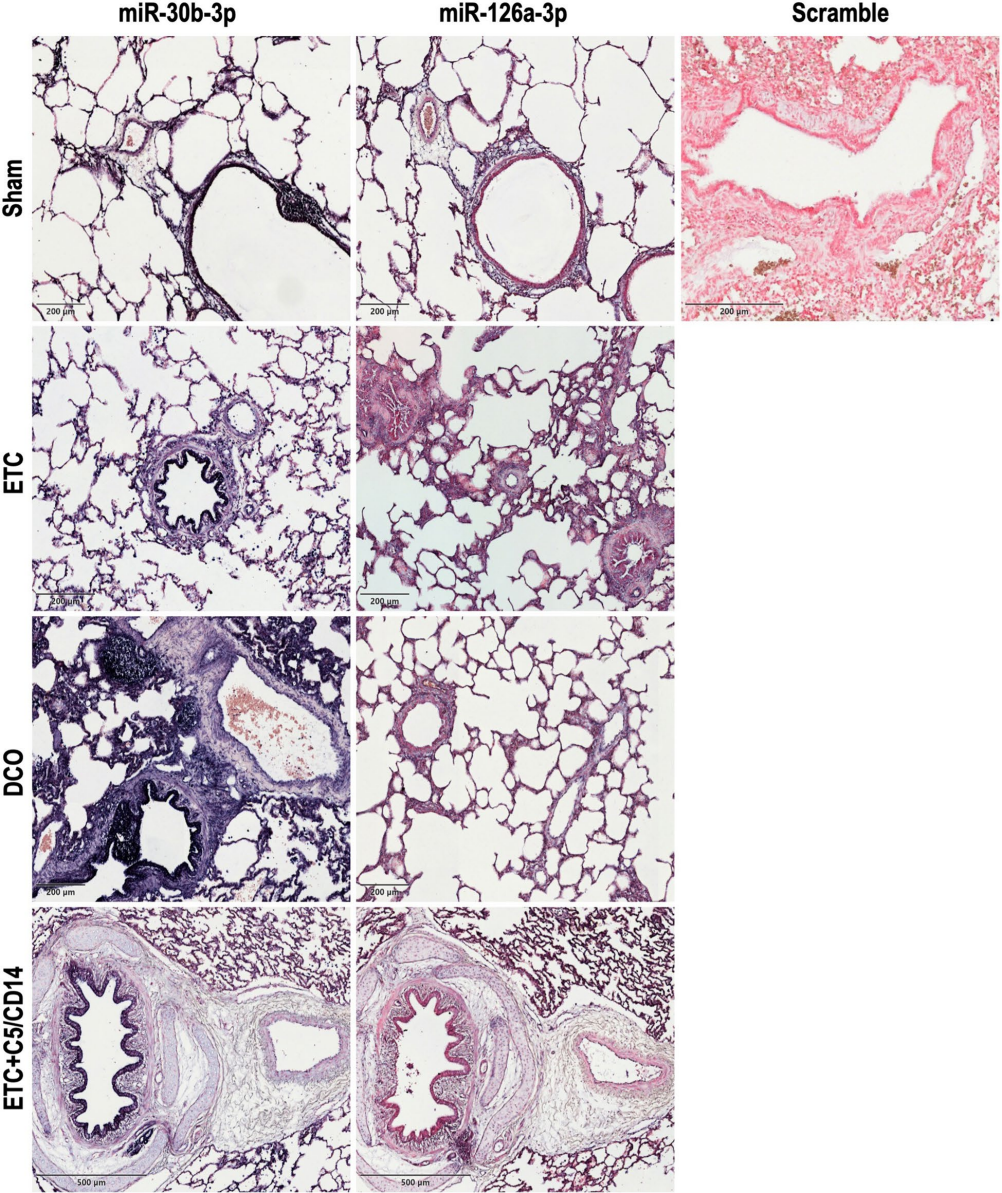
In situ hybridisation of miR-30b-3p and miR-126a-3p for all the investigated groups, i.e., sham (including the scramble control), Early Total Care (ETC), Damage Control Orthopaedics (DCO), and combined ETC + C5/CD14 inhibition drugs. Dark blue = miR-30b-3p, purple = miR-126a-3p, red = counterstaining with fast red

The expression of miR-30b-3p and miR-126a-3p in lung tissue was confirmed with ISH, as depicted in Fig. 5. miR-30b-3p exhibited a higher abundance compared to miR-126a-3p, consistent with the qPCR findings, which indicated that miR-30b-3p was the most upregulated, whereas miR-126a-3p was the most downregulated. Regarding the tissue and cellular distribution of these miRNAs, both similarities and distinct differences were observed. In all treatment groups, both miRNAs displayed similar tissue distributions within the alveoli, with increased localisation around alveolar epithelial cell types −1 and −2. Additionally, both miRNAs were enriched near inflammatory cells; however, miR-30b-3p was predominantly associated with granulocytes, whereas miR-126a-3p was primarily localized near macrophages. Distinct expression patterns were noted in other lung structures. miR-30b-3p exhibited strong localisation along the inner lining of the bronchioles but was scarcely detectable in the surrounding vasculature. In contrast, miR-126a-3p was highly expressed in the vascular endothelium but absent in the respiratory epithelium of the bronchioles. These expression patterns corroborate the qPCR results and highlight the cell type-specific upregulation of distinct miRNAs following blunt chest trauma.

## Discussion

Surgical invasiveness is a well-known contributor to pulmonary complications in polytrauma patients [23], particularly during the early post-traumatic phase. In such cases, these respiratory complications have been shown to directly correlate with increased mortality rates [5]. The present study investigated the expression of 12 miRNAs with known association to pulmonary contusion and inflammation, ARDS, and (blunt) chest trauma in a polytrauma model in pigs. The detected miRNA expression was correlated with two different surgical treatment strategies and a novel immune-modulating drug-based therapy. Relevantly, the porcine model used in this study is highly translational, exhibiting a polytrauma scenario that closely mimics the in-patient situation [24].

We found that miR-21-5p, −23a-3p, −27a-3p, −30b-3p, −126a-3p, −150-3p, −155-5p, −181a-5p, and −223-3p were significantly deregulated in lungs of the polytraumatised animals; their expression specifically correlated to the treatment administered. Reduced surgical invasiveness led to a significant upregulation of anti-inflammatory miRNAs in the DCO group, such as miR-21-5p, −23a-3p, −27a-3p, and −30b-30, all of which exhibit relevant functions in pulmonary inflammation. MiR-21-5p, for example, is an anti-inflammatory miRNA whose downstream impact lies in the suppression of pro-inflammatory factors, such as tumour necrosis factor but also Toll-like receptor signalling [25]. This miRNA is also known to stimulate the expression of IL-10, a potent anti-inflammatory mediator, as well as promoting the polarization of macrophages from the M1- to the M2-phenotype [25]. Both, miR-23a-3p and −27a-3p, have been investigated in murine lung injury models, where it was shown that overexpression of these miRNAs attenuate parenchymal apoptosis and reduce the expression of pro-inflammatory cytokines [26, 27]. Relevantly, the upregulation of miR-30b-3p in the plasma from critically ill septic patients is a known predictor for survival [28]. This indicates its involvement in systemic inflammatory complications and warrants further research in the fields of blunt chest trauma and polytrauma.

Our histology and *in situ* hybridisation results confirmed the miRNA expression findings. It was shown that reduced surgical invasiveness led to the preservation of pulmonary parenchyma. Taken together, these results also match clinical observations in which immune status has proven to be a major factor in clearing patients for definitive fracture surgery [29].

Other two miRNAs, miR-126a-3p and −223-3p, were significantly downregulated in the ETC group, without showing significant deregulations in the DCO group. Research has shown several links between miR-126a-3p and pulmonary pathologies, both acute and chronic, but its function is not yet fully understood. For example, studies on (chronic) respiratory diseases have shown the pro-inflammatory roles of miR-126a-3p. Whereas, a protective role of systemically applied miR-126a-3p mimics on pulmonary function has been demonstrated in a murine model of multiple organ dysfunction [30, 31]. In a different study, Shou et al. showed that the overexpression of miR-126a-3p *in vitro* supported macrophage polarization from M1- to M2-phenotype [32]. These results are in line with those of our ISH experiments, where miR-126a-3p showed the most abundance in the vascular endothelium and around macrophages. Interestingly, miR-223-3p showed a similar expression pattern to that of miR-126a-3p. Both miRNAs have been proposed as biomarkers in several pathologies, including thrombotic risks in myocardial infarction patients [33]. They are both present in platelets in human blood, and the ratio of their expression values can be used to predict endpoints in cardiovascular events [33]. The biological activity of these two miRNAs seems to be also interrelated during impaired respiratory events. MiR-223-3p has shown protective effects on pulmonary inflammation in both *in vitro* and *in vivo* studies. First, by inhibiting several cellular signalling pathways which are key for inflammation, such as the nuclear factor kappa beta and the nucleotide-binding domain, leucine-rich–containing family, pyrin domain–containing-3 inflammasome pathways. Second, and similarly to miR-126a-3p, it actively promotes the polarization of macrophages to the M2-phenotype [34]. The reduced expression of this miRNA may therefore be disadvantageous in the acute post-traumatic phase. However, research in ARDS patients has shown that serum expression levels of miR-223-3p increase significantly between days three and seven of hospitalization, suggesting that the 72 h time of observation after polytrauma in our study may be too early to expect a physiological increase in the expression of this miRNA [35]. Increasing the expression of this miRNA over time, either physiologically or therapeutically, may therefore be beneficial given its multifactorial effect on pulmonary inflammation and regeneration [36].

Overall, the C5/CD14 inhibition drug-based therapy consistently alleviated the effect of enhanced surgical invasiveness in the ETC group as seen from the expression profiles of all detected miRNAs. These results show that the systemic application of this immune-modulating therapy as a novel drug-based approach in the management of polytrauma may be beneficial, influencing local tissue responses after trauma. Our results also demonstrated that the drug successfully addresses relevant target organs. Relevantly, the C5/CD14 inhibition in the ETC group led to miRNA expression levels that were similar to those of the DCO group. Hence, immune modulation, e.g. by C5/CD14 inhibition, may allow for the application of earlier definitive fracture fixation in severely traumatized patients.

Albeit the relevant translational aspect of the porcine polytrauma model used here, this study presents several limitations. The model features a fixed endpoint of 72 h after trauma, thereby excluding possible investigation of miRNA expression kinetics over time. In addition, a small group size was used in the drug-based therapy group. However, the data obtained was revealing and reproducible, guarantying further investigations into the benefits of this drug associated with respiratory performance in polytrauma settings. Lastly, the present model encompasses several components that can contribute to the development of trauma-induced coagulopathy, including major tissue trauma, haemorrhagic shock, subsequent transfusion, and prolonged immobilization. Research from our own group has already shown that increased surgical invasiveness does enhance trauma induced coagulopathy, while the applied drug-based therapy has shown to reduce the thrombo-inflammatory response at the 72 h timepoint [19, 37]. However, future pre-clinical models could entail monitoring of the coagulation system over time to gain more insights into the potential risks of thromboembolic events.

## Conclusion

In this study, the expression of miRNAs with a known relation to respiratory function was demonstrated in lung tissue from polytraumatised pigs. The involvement of miR-21-5p, −23a-3p, −27a-3p, −30b-3p, −126a-3p, −150-3p, −155-5p, −181a-5p, and −223-3p in (patho)physiological responses after blunt chest trauma was demonstrated. Our data indicated that a correlation exists between the expression of these miRNAs in lung tissue with the surgical invasiveness used for fracture stabilization. External fixation resulted in significant upregulations of various anti-inflammatory and tissue regenerative miRNAs in the lung tissue, while intramedullary nailing caused opposing expression dynamics. It was shown that C5/CD14 inhibition drug therapy is beneficial in reducing the risk of pulmonary complications after polytrauma and subsequent surgery. The combination of the C5/CD14 inhibiting drug and the ETC surgical treatment, mitigated the known negative effects of the surgical invasiveness in ETC. Besides regulation of aberrantly expressed miRNAs, the preservation of functional pulmonary parenchyma was shown for this combinatory approach.

In short, this study showed that miRNAs play a vibrant role in chest trauma and that the use of an immune-suppressive therapy may allow for earlier definitive fracture fixation with a better overall outcome. Further research on the long-term effects of immune-modulating therapies in the early post-traumatic phase is required before clinical translation to improve the post-trauma course and outcome.

## Data Availability

No datasets were generated or analysed during the current study.
